# 

**Published:** 1911-03-15

**Authors:** 


					﻿Dental
Register
NELVILLE S. HOFF, Editor,
Ann Arbor, Mich.
SAMUEL A. CROCKER & CO., Publishers
OHIO DENTAL AND SURGICAL DEPOT
35 W. FIFTH STREET, CINCINNATI, O.
PRICE, ONE DOLLAR A YEAR
Entered at the Postoffice at Cincinnati, O., as second-class mail matter.
				

